# Spatiotemporally synchronized dual-wavelength measurement of aerosol optical properties using a dual-differential sphere–tube coupled photoacoustic–scattering system

**DOI:** 10.1016/j.pacs.2026.100864

**Published:** 2026-07-29

**Authors:** Zhengang Li, Hepeng Zhang, Daming Dong, Yonghua Fang

**Affiliations:** a Key Laboratory of Environmental Optics and Technology, Anhui Institute of Optics and Fine Mechanics, Hefei Institutes of Physical Science, Chinese Academy of Sciences, Hefei 230031, China; b University of Science and Technology of China, Hefei 230026, China; c Research Center of Intelligent Equipment for Agriculture, Beijing Academy of Agriculture and Forestry Sciences, Beijing 100097, China

**Keywords:** Photoacoustic spectroscopy, Integrating-sphere nephelometry, Aerosol optical properties, Dual-differential acoustic resonance, Spatiotemporal synchronization

## Abstract

To reduce potential inconsistencies in particle transport, temporal response, and microenvironment in conventional split-type aerosol absorption–scattering systems, we developed a dual-wavelength spatiotemporally synchronized measurement method. Two integrating spheres serve as scattered-light collection cavities and acoustic buffers and form a low-frequency differential Helmholtz resonator, while the central tube supports a high-frequency differential second-order longitudinal mode. The 450 and 532 nm lasers were modulated at 2572 and 169 Hz and synchronously demodulated using an FPGA-based digital lock-in amplifier. Absorption channels were calibrated with NO₂, and scattering channels with N₂/CO₂ Rayleigh scattering. At 1 s integration and a 3*σ* criterion, the 532 nm absorption and scattering limits of detection were 1.2 and 1.32 Mm⁻¹ . Water-mist, controlled-combustion, and field tests evaluated channel isolation, relative spectral response, and ambient-operation feasibility. The system simultaneously measures dual-wavelength absorption and scattering within a common volume through a single sampling path.

## 1 Introduction

The aerosol absorption coefficient ($b_{\text{abs}}$) and scattering coefficient ($b_{\text{sca}}$) are key parameters for evaluating aerosol radiative effects, and together they determine the single-scattering albedo (SSA) [1], [2], [3], [4], [5], [6], [7], [8]. The wavelength dependences of absorption and scattering are commonly described by the absorption Ångström exponent (AAE) and scattering Ångström exponent (SAE), respectively:

(1) $$b_{\text{abs}}(\lambda )\;=\;K_{\text{abs}}\lambda^{-\text{AAE}}$$

$$b_{\text{sca}}(\lambda )\;=\;K_{\text{sca}}\lambda^{-\text{SAE}}$$

where $K_{\text{abs}}$ and $K_{\text{sca}}$ are proportionality constants. AAE is primarily influenced by particle chemical composition and mixing state. Black carbon generally exhibits weak wavelength-dependent absorption, with an AAE close to 1, whereas brown carbon shows stronger absorption in the near-ultraviolet and short-wavelength visible regions. SAE is mainly affected by particle size distribution, morphology, and complex refractive index; a larger contribution from fine particles generally produces stronger short-wavelength scattering enhancement [9].

The wavelengths of 450 and 532 nm were selected in this study. The 450 nm channel is relatively sensitive to components exhibiting enhanced short-wavelength absorption, whereas 532 nm is commonly used as a reference wavelength in the visible region. For two discrete wavelengths, *λ*_1_ and *λ*_2_, the two-point AAE and SAE are calculated as

(2) $${\text{AAE}}_{\lambda_{1}/\lambda_{2}}\;=\;-\frac{\ln\left[b_{\text{abs}}(\lambda_{1})/b_{\text{abs}}(\lambda_{2})\right]}{\ln\left(\lambda_{1}/\lambda_{2}\right)}$$

$${\text{SAE}}_{\lambda_{1}/\lambda_{2}\;}=\;-\frac{\ln\left[b_{\text{sca}}(\lambda_{1})/b_{\text{sca}}(\lambda_{2})\right]}{\ln\left(\lambda_{1}/\lambda_{2}\right)}$$

Hereafter, AAE and SAE specifically refer to AAE_450/532_ and SAE_450/532_. These two-point indices describe only the average wavelength dependence over 450–532 nm and should not be interpreted as fitted exponents over a broader spectral range.

In addition to AAE, SAE, and SSA, multi-wavelength absorption and scattering coefficients, when combined with independently measured particle size distributions, can provide optical constraints for retrieving aerosol complex refractive index, mixing state, and absorption-enhancement characteristics [10], [11], [12], [13]. When further combined with chemical-composition and toxicological measurements, these optical parameters may also support investigations of the microphysical properties and potential health effects of source-emitted aerosols [14], [15]. Nevertheless, the dual-wavelength optical parameters measured in the present study alone are insufficient to uniquely retrieve the complex refractive index or determine particle toxicity.

Filter-based absorption instruments, such as the AE33 and PSAP, and integrating nephelometers are widely used for aerosol optical monitoring [16], [17], [18], [19], [20], [21]. Filter-based absorption measurements may be affected by loading effects and multiple scattering within the filter substrate, whereas nephelometers are subject to angular truncation errors. Photoacoustic spectroscopy (PAS) enables in situ measurement of optical absorption by gases and suspended particles, while integrating-sphere scattering detection improves the collection efficiency of multidirectional scattered photons [22], [23], [24], [25], [26], [27], [28], [29], [30], [31], [32], [33], [34], [35], [36], [37]. The two techniques are therefore complementary. Recent advances in photoacoustic and related indirect-absorption spectroscopy have further improved sensing performance through cascaded acoustic resonance, differential resonant configurations, and intelligent algorithm-assisted signal analysis [33], [34], [35].

However, conventional split-type configurations generally employ separate sampling lines, measurement cells, and response times, which may introduce inconsistencies in particle transport, temperature and relative humidity, and temporal response. Such discrepancies can affect the synchronous calculation of absorption, scattering, and their derived optical parameters. Simultaneous measurements within a common sampling volume are therefore practically valuable.

Multi-wavelength measurements are commonly implemented using either parallel multi-cell configurations or time-division multiplexing within a single cell. Parallel configurations enable simultaneous detection but increase system size and complexity, whereas time-division multiplexing facilitates integration at the expense of temporal consistency among different wavelength channels [38], [39]. Frequency-division multiplexing allows multiple modulated light sources to operate simultaneously, but requires spectrally separated acoustic modes within the same gas cell to reduce interchannel crosstalk.

The integration of PAS and integrating-sphere scattering detection is not introduced for the first time in this study. Our previous work [29] demonstrated combined absorption and scattering measurements at 532 nm, while Wang et al. [19] reported four-wavelength absorption and scattering measurements within a common sample volume. The objective of the present study is neither to maximize the number of detection wavelengths nor to achieve the lowest single-channel detection limit. Instead, the two integrating spheres are used simultaneously as scattered-light collection cavities, acoustic buffer chambers, and components of a low-frequency differential Helmholtz resonator, while the central acoustic tube supports a high-frequency differential second-order longitudinal standing-wave mode. The 450 and 532 nm photoacoustic signals are carried by two spectrally separated differential acoustic modes and synchronously demodulated using an FPGA-based digital lock-in amplifier. This architecture enables dual-wavelength absorption and scattering measurements through a single sampling path and within a common measurement volume, thereby providing a structural basis for reducing potential channel-to-channel discrepancies associated with separate sampling lines and measurement cells.

In this study, the two acoustic modes and four measurement channels were first characterized and calibrated, and the zero-point stability and detection limits of the representative 532 nm channels were evaluated. Water-mist, controlled-combustion, and field experiments were then conducted to assess channel isolation, relative dual-wavelength absorption responses, and the feasibility of continuous operation under ambient conditions, respectively.

## 2 Structural design and finite-element analysis of the dual-differential sphere–tube coupled gas cell

### 2.1 Gas-cell function sharing and differential configuration

**Fig. 1:**
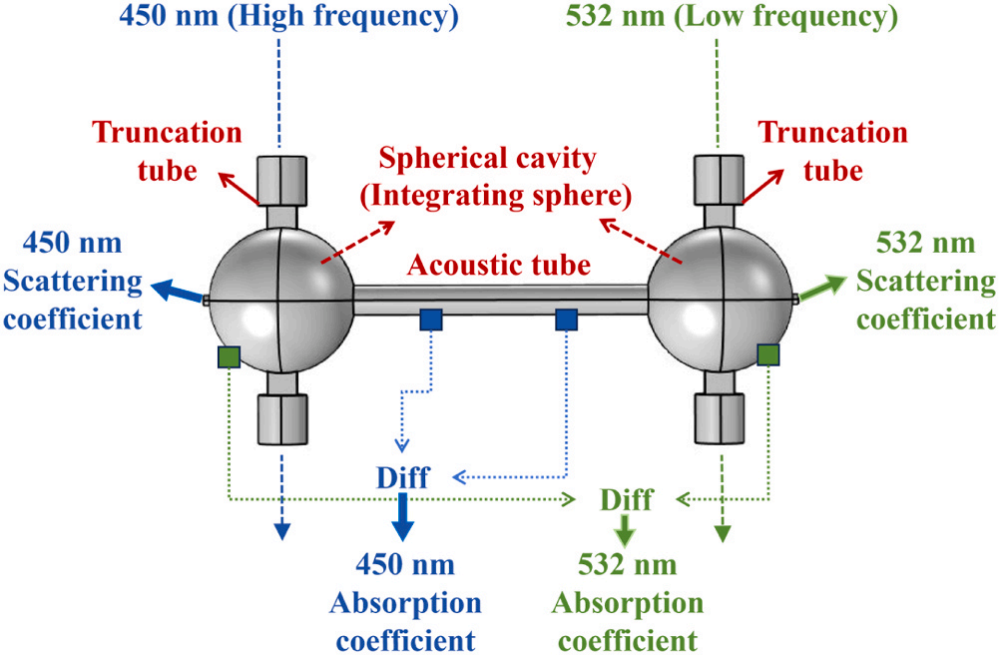
Core structure of the dual-differential sphere–tube coupled gas cell.

To reduce potential channel-to-channel discrepancies associated with separate sampling lines, independent measurement cells, and asynchronous responses in conventional split-type absorption–scattering systems, a spatiotemporally synchronized dual-differential sphere–tube coupled gas cell was designed. As shown in Fig. 1, the gas cell consists of a central acoustic tube, two symmetrically arranged integrating spheres, and peripheral truncation tubes. Aerosol samples enter the common measurement volume through a single inlet, where the 450 and 532 nm absorption and scattering signals are acquired on a common time base. This configuration combines optical collection and acoustic resonance functions within a single structure.

From the optical perspective, the inner surfaces of the spherical cavities are coated with highly reflective expanded polytetrafluoroethylene (PTFE), forming integrating-sphere nephelometers for collecting scattered photons over a broad angular range. From the acoustic perspective, under high-frequency excitation, the spherical cavities have much larger volumes than the central resonant tube and approximately act as zero-pressure terminations of an open tube, thereby serving as acoustic buffer chambers for an H-type photoacoustic cell. Under low-frequency excitation, the same spherical cavities provide the acoustic compliance of a differential Helmholtz resonator. This multifunctional configuration increases system integration and allows absorption and scattering measurements to share a common measurement volume.

The optical and acoustic functions of the gas cell are not fully independent. The highly reflective inner surfaces enhance scattered-light collection but also cause multiple diffuse reflections of the excitation beams. Non-axial stray photons incident on the microphone diaphragms, metallic cell walls, or quartz windows may generate coherent photothermal backgrounds and affect the limits of detection of the photoacoustic channels. In addition, the truncation tubes used to reduce scattering-light loss through the sphere apertures alter the external acoustic impedance and may consequently affect the resonance quality factor. A spatially symmetric differential configuration and finite-element analysis were therefore employed to balance scattering-collection efficiency and photoacoustic resonance performance.

### 2.2 Optical design of the integrating-sphere nephelometers and truncation-error compensation

An ideal integrating-sphere nephelometer has a completely enclosed spherical surface. In practice, however, apertures are required for axial laser transmission, gas delivery, microphone installation, and photomultiplier-tube (PMT) detection. These apertures allow scattered photons to escape and introduce both aperture loss and angular truncation error.

#### 2.2.1 Integrating-sphere aperture fraction and optical multiplication factor

The integrating sphere has an inner radius of $R_{\text{s}}=30\text{ mm}$ and an inner surface area of $A_{\text{sphere}}=4\text{π}R_{\text{s}}^{2}\text{≈}11309.7\;{\text{mm}}^{2}$. Each sphere contains five apertures: one acoustic-tube coupling aperture with a radius of $r_{\text{p}}=5\text{ mm}$, two axial laser apertures with a radius of $r_{h}\text{=}2.5\text{ mm}$, one PMT detection aperture with a radius of $r_{\text{pmt}}=4\text{ mm}$, and one microphone aperture with a radius of $r_{\text{m}}=3\text{ mm}$. The gas inlet and outlet were positioned on the outer truncation tubes to avoid additional openings in the sphere walls. The total effective aperture area is $A_{\text{holes}}\text{≈}196.3\;{\text{mm}}^{2}$, giving an aperture fraction of 1.7%, which is below the commonly recommended 5% limit for integrating-sphere designs [40]. The theoretical optical multiplication factor is expressed as

(3) $$M=\frac{\rho }{1-\rho (1-f)}$$

where ρ is the diffuse reflectance of the inner coating. For an expanded PTFE coating with ρ=99%, the calculated multiplication factor is approximately 36.9, indicating efficient accumulation of weak aerosol-scattering signals within the sphere.

#### 2.2.2 Geometrical correction of the truncation angle and volume compensation

**Fig. 2:**
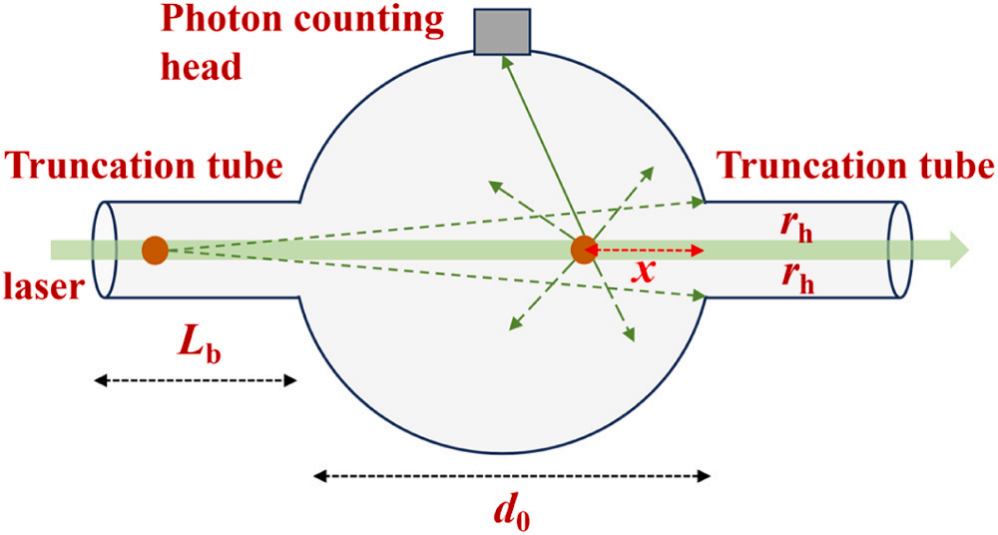
Schematic of the truncation-tube structure of the integrating sphere.

Angular truncation is a systematic source of uncertainty in integrating-nephelometer measurements [18]. Owing to the finite optical apertures, some light scattered in the near-forward (0°–10°) and near-backward (170°–180°) directions can escape without undergoing diffuse reflection at the sphere wall. Without a corrective structure, particles at different axial positions subtend different solid angles at the apertures, resulting in a nonuniform spatial response. To reduce this effect, axial truncation tubes with a length of $L_{\text{b}}=30\;\text{mm}$ were installed at the laser entrance and exit apertures, as shown in Fig. 2.

The maximum truncation angle is estimated using the diameter of the circular optical aperture,$\;D_{\text{diag}}=\;2r_{h}\;=\;5\;\text{mm}$. For the original spherical geometry without truncation tubes, the axial distance between a particle and the exit aperture is denoted by x, where 0≤x≤60mm. The position-dependent truncation angle is

(4) $$\theta (x)=\arctan\frac{D_{\text{diag}}}{2x}$$

For a particle located at the sphere center (x=30mm), the calculated truncation angle is 4.8°. As the particle approaches the aperture (x→0), θ(x) increases rapidly toward 90°, leading to both signal loss and a nonuniform spatial response.

The two truncation tubes extend the particle-containing region along the optical axis and provide a complementary sampling volume. Scattering losses from particles inside the sphere can therefore be partly compensated by forward-scattered light from particles within the inlet truncation tube that enters the sphere. Within the effective compensation interval $0\leq x\leq L_{\text{b}}$, the equivalent corrected truncation angle is

(5) $$\theta_{\text{c}}(x)=\arctan\left(\frac{D_{\text{diag}}}{2(x+L_{\text{b}})}\right)$$

Using $D_{\text{diag}}=\;5\;\text{mm}$ and $L_{\text{b}}=30\;\text{mm}$, the geometrically estimated effective truncation angle ranges from 2.4° to 4.8°, which is smaller than the reported physical truncation-angle range of 7°–15° for the TSI 3563. Because the two instruments differ in structure and in the definition of truncation angle, this comparison is intended only as an order-of-magnitude reference.

### 2.3 Dual-differential acoustic modes and frequency-division multiplexing

To synchronously extract the two photoacoustic signals generated by the 450 and 532 nm lasers within the same sphere–tube coupled gas cell, two well-separated acoustic modes—a differential second-order longitudinal standing-wave mode and a differential Helmholtz mode—were used to implement frequency-division multiplexing. The two lasers were intensity-modulated at their respective acoustic resonance frequencies, and the differential microphone signals were synchronously demodulated at the two reference frequencies using an FPGA-based digital lock-in amplifier. The frequency separation between the two acoustic modes helps reduce crosstalk between the two wavelength channels.

For the high-frequency channel, the central acoustic tube supports a second-order longitudinal standing-wave mode. The acoustic pressure is primarily concentrated inside the tube, with two approximately anti-phase pressure antinodes located near *L*/4 and 3 *L*/4 along the tube axis. A pair of microphones installed at these symmetric positions is used for differential detection. The differential operation causes the two anti-phase photoacoustic responses to add while reducing in-phase common-mode disturbances associated with mechanical vibration and gas flow. This mode is used to detect the 450 nm photoacoustic signal.

For the low-frequency channel, the gas column in the central connecting tube acts as an equivalent acoustic mass, while the two integrating-sphere cavities provide acoustic compliance. Together, they form a dual-cavity coupled differential Helmholtz resonator. At resonance, the acoustic pressures in the two integrating spheres are approximately anti-phase. A microphone pair installed at symmetric positions in the two spheres therefore enables differential detection, combining the anti-phase target responses while reducing common-mode disturbances. This mode is used to detect the 532 nm photoacoustic signal.

The two modes are clearly separated in both resonance frequency and spatial acoustic-field distribution and can therefore be simultaneously excited and independently demodulated at different modulation frequencies. Compared with sequential wavelength switching, this frequency-division scheme is advantageous for maintaining temporal consistency between wavelength-dependent parameters during rapidly changing aerosol measurements.

### 2.4 Finite-element validation of the dual-differential acoustic modes

A thermoviscous-acoustics finite-element model was developed to verify the acoustic modes and the selected differential-detection positions in the sphere–tube coupled gas cell. The model included the central acoustic tube, the two integrating spheres, the truncation tubes, and the actual aperture structures. Air was used as the acoustic medium at a temperature of 293.15 K and a pressure of 1 atm. Considering the optical-aperture requirements of the scattering channels, the integrating-sphere dimensions, and the required frequency separation between the two modes, the effective length and inner radius of the central acoustic tube were set to 12 cm and 5 mm, respectively, and the inner radius of each integrating sphere was set to 30 mm. Eigenfrequency analysis yielded resonance frequencies of 151.46 Hz for the differential Helmholtz mode and 2369.7 Hz for the second-order longitudinal standing-wave mode.

**Fig. 3:**
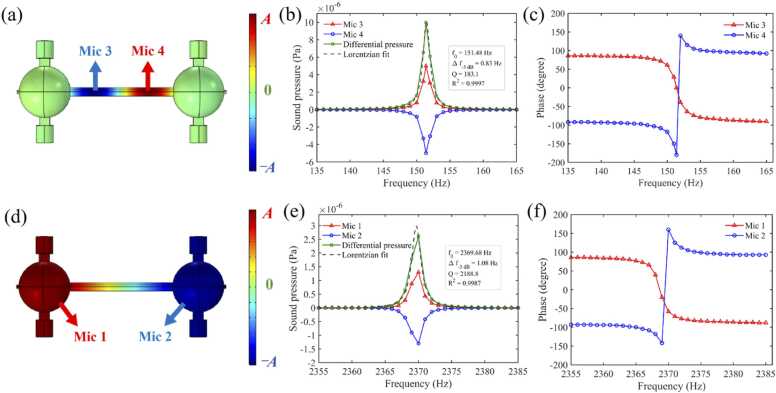
Finite-element results for the dual-differential acoustic modes: (a) acoustic-pressure field of the second-order longitudinal standing-wave mode; (b) amplitude–frequency response; (c) phase–frequency response; (d) acoustic-pressure field of the differential Helmholtz mode; (e) amplitude–frequency response; and (f) phase–frequency response.

As shown in Fig. 3(a)–(c), near 2369.7 Hz, the acoustic pressure is concentrated primarily in the central tube, with two anti-phase pressure antinodes located near *L*/4 and 3 *L*/4. The phase difference between the two detection positions approaches 180∘ near resonance. As shown in Fig. 3(d)–(f), near 151.46 Hz, anti-phase acoustic-pressure responses are formed in the two integrating spheres, while the gas column in the central tube oscillates between the two cavities. These results verify the spatially symmetric conditions required for differential detection and demonstrate clear separation of the two modes in both acoustic-field distribution and resonance frequency.

The differential acoustic-pressure responses obtained from the two detection positions were fitted using Lorentzian functions. Because the ordinates in Fig. 3, Fig. 6 represent acoustic-pressure or photoacoustic-signal amplitude rather than power, the −3 dB frequencies were defined using the amplitude criterion. After subtraction of the fitted baseline Y₀, the two −3 dB frequencies f₁ and f₂ were determined from $20\log_{10}\left\{\left[Y(f)-Y_{0}\right]/\left[Y(f_{0})-Y_{0}\right]\right\}\;=\;-3$, which is equivalent to $Y(f_{1,2})-Y_{0}=\left[Y(f_{0})-Y_{0}\right]/\sqrt{2}\text{≈}0.707\left[Y(f_{0})\right]$. The −3 dB bandwidth and quality factor were then calculated as $\text{Δ}f_{3\text{dB}}\;={\;f}_{2}-f_{1}$and $Q\;=\;f_{0}/\text{Δ}f_{3\text{dB}}$, respectively. Therefore, the visually wider half-height width of the plotted amplitude response corresponds to approximately −6.02 dB rather than −3 dB. For the differential Helmholtz mode, the fitted center frequency, −3 dB bandwidth, and quality factor were approximately 151.48 Hz, 0.83 Hz, and 183, respectively. For the differential second-order longitudinal standing-wave mode, the corresponding values were approximately 2369.68 Hz, 1.08 Hz, and 2190. The spatial and frequency separation of these two modes provides the acoustic basis for synchronous frequency-division excitation and independent demodulation of the 450 and 532 nm photoacoustic signals.

## 3 System construction and calibration experiments

### 3.1 System construction

**Fig. 4:**
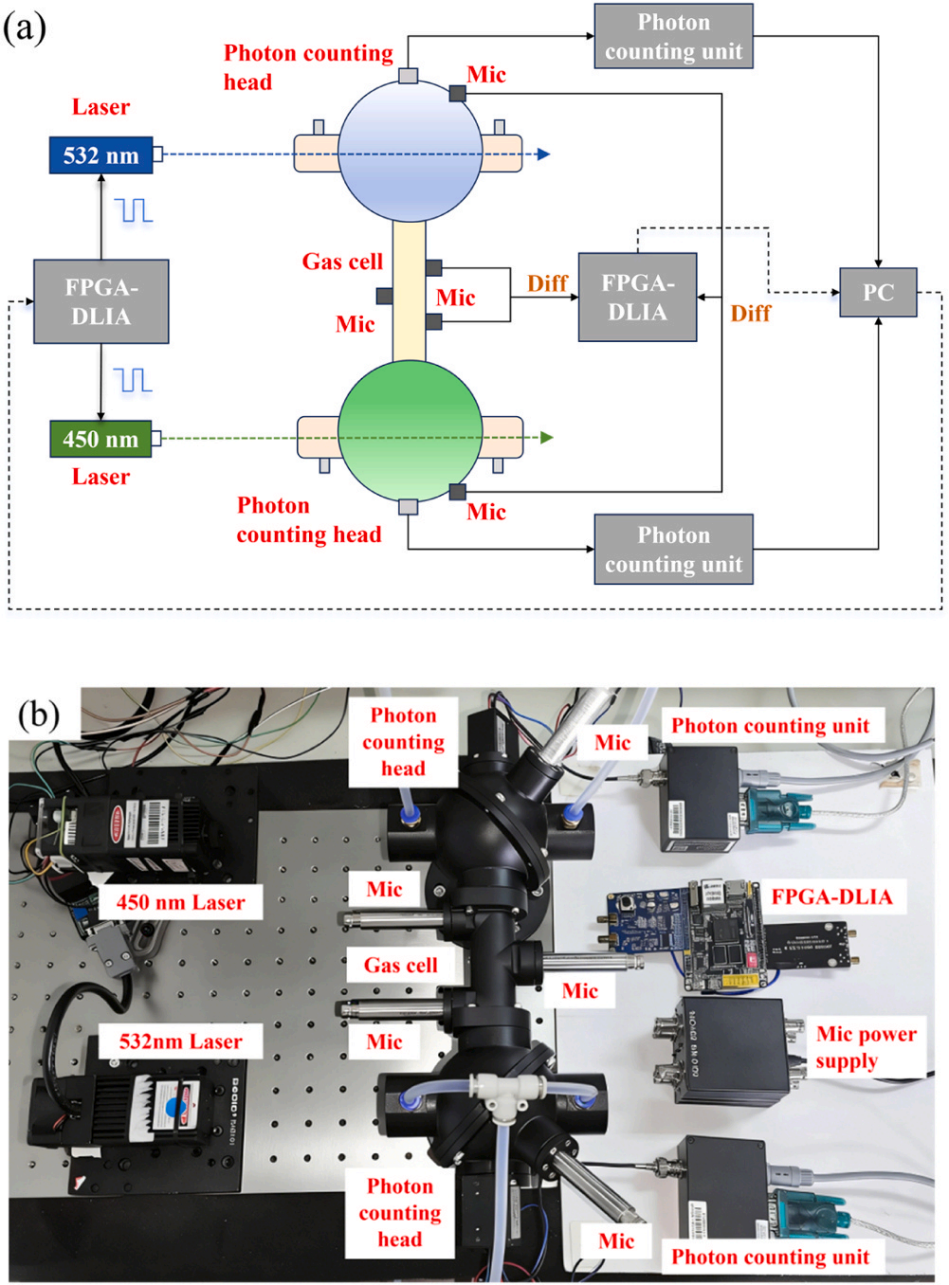
Dual-wavelength system for spatiotemporally synchronized measurement of aerosol optical properties: (a) overall system configuration and (b) photograph of the experimental setup.

The gas cell was fabricated and integrated with the optical and electronic components using the structural parameters determined in Section 2. The overall system configuration and a photograph of the experimental setup are shown in Fig. 4.

**Fig. 5:**
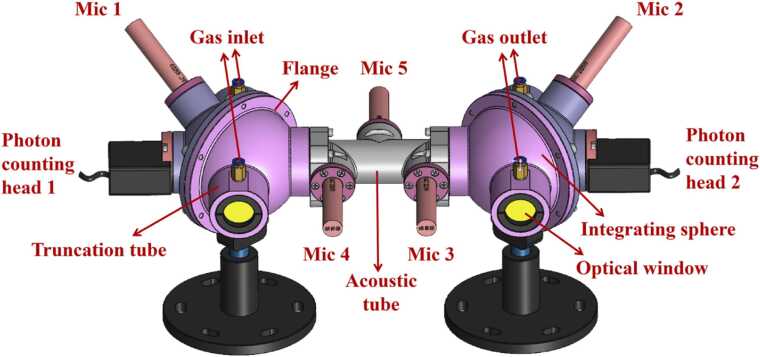
Mechanical structure of the sphere–tube coupled gas cell.

The mechanical structure of the gas cell is shown in Fig. 5. The gas-cell housing was machined from 6061 aluminum alloy, and its inner surface was black-anodized to reduce photothermal backgrounds caused by non-axial stray light. Each integrating sphere contains a removable expanded-PTFE liner with high diffuse reflectance in the visible region, facilitating inspection and maintenance during extended operation. Potential changes in reflectance and background response caused by particle deposition, together with the corresponding maintenance procedures, are discussed in Section 4.4.

Two microphones were installed near the *L*/4 and 3 *L*/4 positions of the central acoustic tube, corresponding to the two anti-phase pressure antinodes of the second-order longitudinal standing-wave mode. Two additional microphones of the same type were installed at spatially symmetric positions in the integrating spheres for detection of the low-frequency differential Helmholtz mode. Mic 5 is a reserved microphone port and was not used in the present experiments. When the signals from each microphone pair have approximately equal amplitudes and opposite phases, differential processing combines the target photoacoustic responses while suppressing in-phase disturbances coupled to both detection positions. The gas inlet and outlet were located on the two truncation tubes. The symmetric gas-flow arrangement reduces stagnant regions within the cell, and sealing rings were installed at the mechanical interfaces to maintain airtightness.

A 532 nm solid-state laser (Changchun New Industries, PGL-V-H−532) and a 450 nm diode laser (Dongguan Lanyu, K40B50W; maximum output power, 4.5 W) were used as the excitation sources. The two lasers were intensity-modulated at their respective acoustic resonance frequencies. Photoacoustic signals were detected using SAC2250 prepolarized measurement microphones with a sensitivity of 50 mV Pa^−1^. Scattered-light signals at the two wavelengths were collected by H10682–110 photon-counting heads installed at the integrating-sphere detection ports and recorded using CH297 photon-counting units.

The laser modulation signals and quadrature demodulation of the differential photoacoustic signals were generated and implemented using a custom-developed FPGA-based digital lock-in amplifier (FPGA-DLIA). Its circuit design and independent performance characterization have been reported previously [41].

### 3.2 Quantitative calibration and detection-sensitivity evaluation

The system outputs differential photoacoustic amplitudes and PMT photon count rates at 450 and 532 nm. The photoacoustic amplitudes were converted to absorption coefficients through NO_2_ calibration, whereas the photon count rates were converted to scattering coefficients using two-point N_2_/CO_2_ Rayleigh-scattering calibration. The calibrated data were subsequently used to calculate AAE_450/532_, SAE_450/532_, and SSA on a common time base.

#### 3.2.1 Acoustic response and NO_2_ calibration of the dual-wavelength photoacoustic channels

**Fig. 6:**
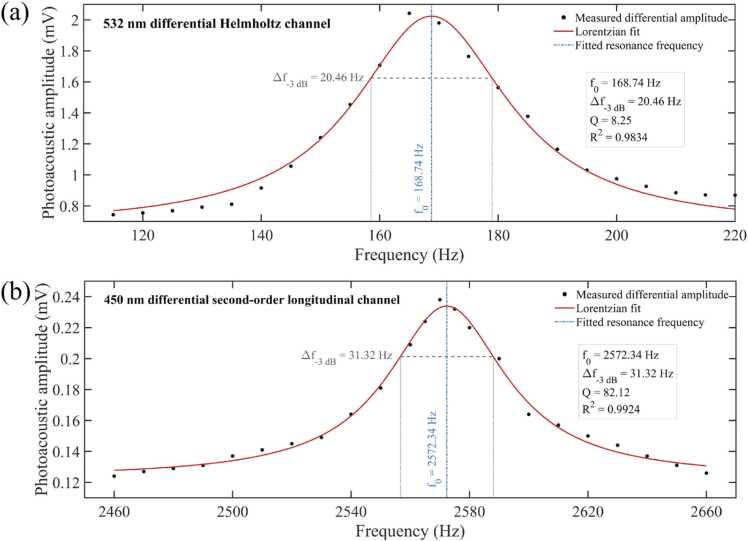
Experimental frequency sweeps and Lorentzian fits of the dual-differential acoustic modes: (a) 532 nm differential Helmholtz mode and (b) 450 nm differential second-order longitudinal standing-wave mode.

The resonance frequencies and bandwidths of the fabricated gas cell may deviate from the ideal finite-element results because of machining tolerances, microphone insertion depth, flange assembly, and the acoustic properties of the actual gas medium. To experimentally characterize the resonance frequency, linewidth, and quality factor of each differential acoustic mode, 10 ppm NO_2_ standard gas was introduced into the cell at a constant flow rate. Frequency sweeps were performed over 110–220 Hz and 2460–2660 Hz for the low- and high-frequency modes, respectively. At each frequency, the outputs of the spatially symmetric microphone pairs were differentially processed and demodulated using the FPGA-DLIA. The measured responses and Lorentzian fits are shown in Fig. 6. The experimental −3 dB bandwidths and *Q* factors were determined using the same baseline-corrected amplitude criterion defined in Section 2.4.

As summarized in Table 1, the experimental quality factor of the high-frequency second-order longitudinal mode was approximately ten times that of the low-frequency Helmholtz mode. The high-frequency mode therefore exhibited greater frequency selectivity but was also more sensitive to modulation-frequency drift. Relative to the finite-element results, the measured resonance frequencies of the differential Helmholtz and second-order longitudinal modes were shifted upward by 11.4% and 8.55%, respectively, and the experimental resonance peaks were substantially broader. These differences may arise from machining and assembly tolerances, acoustic loading introduced by the microphones, leakage through gas-flow apertures, and damping associated with continuous gas flow, which were not fully represented in the finite-element model. Because the individual contributions of these factors were not independently quantified, the measured resonance frequencies were used as the laser modulation frequencies in the subsequent experiments.

**Table 1:** Comparison of the finite-element and experimental parameters of the dual-differential acoustic modes.

**Acoustic mode**	**Data source**	***f***_**0**_**(Hz)**	**Δ*****f***_**−3dB**_**(Hz)**	***Q***	***R***^**2**^
Differential Helmholtz mode	Finite element	151.48	0.83	183	—
	Experiment	168.74	20.46	8.25	0.9834
Differential second-order longitudinal mode	Finite element	2369.68	1.08	2190	—
	Experiment	2572.34	31.32	82.12	0.9924

Accordingly, the modulation frequencies of the 450 and 532 nm lasers were set to 2572 and 169 Hz, respectively. A dynamic gas-mixing system was used to dilute a 10 ppm NO_2_ standard gas with high-purity N_2_, producing five concentration levels of 0.1, 0.5, 1.0, 1.5, and 2.0 ppm. The reference absorption coefficient at wavelength λ was calculated as

(6) $$b_{\text{abs},\text{λ}}^{\text{ref}}\;=\;N_{{\text{NO}}_{2}}\sigma_{{\text{NO}}_{2},\text{λ}}$$

where $N_{{\text{NO}}_{2}}$ is the NO_2_ molecular number density under the calibration conditions of 25℃ and 1 atm, and $\sigma_{{\text{NO}}_{2}}$ is the absorption cross-section at wavelength λ.The absorption cross-sections used at 450 and 532 nm were 4.2 × 10^−19^ and 1.5 × 10^−19^ cm^2^molecule^−1^, respectively, based on the HITRAN database.

The steady-state differential photoacoustic amplitude was linearly fitted against the calculated reference absorption coefficient:

(7) $$V_{\text{PA},\text{λ}}\;=\;S_{\text{abs},\text{λ}}\;b_{\text{abs},\text{λ}}^{\text{ref}}+V_{0,\text{λ}}$$

where $V_{\text{PA},\text{λ}}$ is expressed in mV, $b_{\text{abs},\text{λ}}^{\text{ref}}$ in Mm^−1^, $S_{\text{abs},\text{λ}}$ is the absorption-response slope, and $V_{0,\text{λ}}$ is the fitted intercept. The calibration relationships for the two channels were

$$V_{\text{PA},450}\text{ = }1.417\text{×}10^{-4}\;b_{\text{abs},450}\text{+}0.0097\text{, }R^{2}\;\text{= }0.9985$$

(8) $$V_{\text{PA},532}\text{ = }1.407\text{×}10^{-3}\;b_{\text{abs},532}\text{+}0.0924\text{,}{\;R}^{2}\;\text{= }0.9997$$

The corresponding absorption-response slopes were 0.142 and 1.407 *μ*V/(Mm⁻¹) for the 450 and 532 nm channels, respectively. The absorption coefficient of an unknown sample was calculated as

(9) $$b_{\text{abs},\text{λ}}\;=\;\frac{V_{\text{PA},\text{λ}}-V_{0,\text{λ}}}{S_{\text{abs},\text{λ}}}$$

**Fig. 7:**
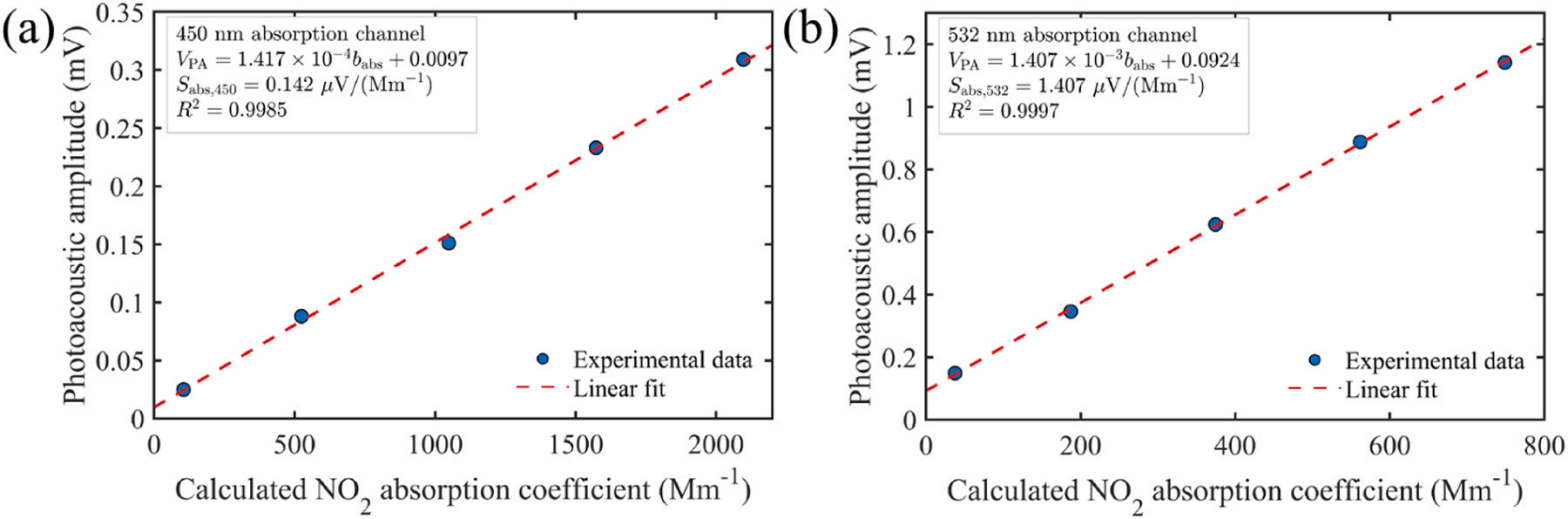
NO_2_ calibration results for the dual-wavelength absorption channels: (a) 450 nm channel and (b) 532 nm channel.

The calibration results are shown in Fig. 7.

#### 3.2.2 Two-point N_2_/CO_2_ Rayleigh-scattering calibration of the dual-wavelength scattering channels

The 450 and 532 nm scattering channels were calibrated using a two-point N_2_/CO_2_ Rayleigh-scattering method. Dry high-purity N_2_ and CO_2_ were introduced sequentially into the gas cell. After the photon count rates stabilized, the mean count rate over a continuous 2 min interval was recorded for each channel and denoted by $C_{{\text{N}}_{2},\text{λ}}$ and $C_{{\text{CO}}_{2},\text{λ}}$, respectively. Using the theoretical Rayleigh-scattering coefficients of the two gases at the same temperature and pressure, the conversion factor was defined as

(10) $$K_{\text{sca},\text{λ}}\;=\;\frac{b_{\text{sca},{\text{CO}}_{2},\text{λ}}-b_{\text{sca},{\text{N}}_{2},\text{λ}}}{C_{{\text{CO}}_{2},\text{λ}}-C_{{\text{N}}_{2},\text{λ}}}$$

where $b_{\text{sca},{\text{N}}_{2},\text{λ}}$ and $b_{\text{sca},{\text{CO}}_{2},\text{λ}}$ are the theoretical Rayleigh-scattering coefficients of N_2_ and CO_2_, respectively. The scattering coefficient of an unknown sample was then calculated as

(11) $$b_{\text{sca},\text{λ}}(t)\;=\;b_{\text{sca},{\text{N}}_{2},\text{λ}}+K_{\text{sca},\text{λ}}[C_{\text{λ}}(t)-C_{{\text{N}}_{2},\text{λ}}]$$

Provided that the dark-count and residual stray-light backgrounds remain stable between the two calibration measurements, this two-point differential form subtracts the fixed background offset. The resulting conversion factors were 0.00105 and 0.00225 Mm⁻¹ /(count·s⁻¹) for the 450 and 532 nm channels, respectively.

#### 3.2.3 Zero-point stability and limits of detection of the 532 nm channels

A continuous 1 h zero-gas test was performed only for the 532 nm absorption and scattering channels. These data were therefore used to demonstrate the calculation of the limits of detection based on zero-point fluctuations and a 3*σ* criterion. Because the two wavelength channels differ in laser power, acoustic mode, quality factor, and response coefficient, the resulting limits of detection were not extrapolated to the 450 nm channels.

**Fig. 8:**
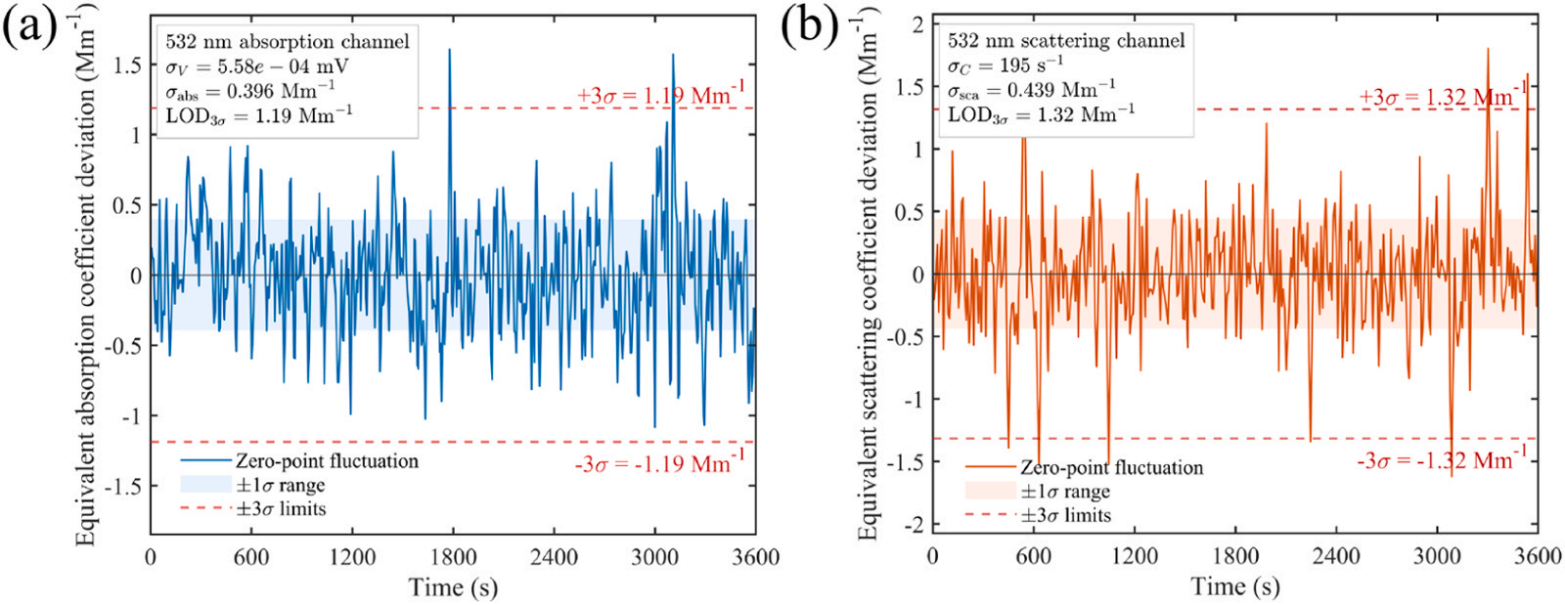
Zero-point stability and limits of detection of the 532 nm channels: (a) equivalent absorption-coefficient deviation and (b) equivalent scattering-coefficient deviation.

Under constant-temperature laboratory conditions, the sample gas was dried using a silica-gel diffusion dryer, and high-purity N_2_ was continuously introduced into the cell at 500 mL/min. The integration time was 1 s, and the test duration was 1 h. The mean values of the absorption signal and scattering count-rate time series were subtracted, and the residuals were converted to equivalent optical-coefficient deviations using the corresponding calibration factors. The results are shown in Fig. 8.

The standard deviation of the 532 nm photoacoustic amplitude was 5.58 × 10^−4^ mV. Using the absorption-response slope of 1.407 × 10^−3^ mV/(Mm^−1^), the equivalent absorption-coefficient standard deviation was

(12) $$\sigma_{\text{abs}}\;=\;\frac{\sigma_{V}}{S_{\text{abs},532}}\text{ = }0.396\;{\text{Mm}}^{-1}$$

The corresponding 3*σ* limit of detection was 1.19 Mm^−1^, reported as approximately 1.2 Mm^−1^.

For the 532 nm scattering channel, the mean N_2_ photon count rate was approximately 9480 s^−1^, with a standard deviation of 195 s^−1^. Using the conversion factor of 0.00225 Mm⁻¹ /(count·s⁻¹), the equivalent scattering-coefficient standard deviation was

(13) $$\sigma_{\text{sca}}\;={\;K}_{\text{sca},532}\sigma_{C}\text{ = }0.439\;{\text{Mm}}^{-1}$$

The corresponding 3*σ* limit of detection was 1.32 Mm^−1^.

## 4 Results and discussion

### 4.1 Channel-isolation test under a strong-scattering perturbation

To evaluate whether a strong scattering perturbation produces a discernible synchronous response in the photoacoustic absorption channel, a high concentration of water-mist droplets generated by an ultrasonic humidifier was continuously introduced into the gas cell. The water mist was used to simulate a strongly scattering disturbance with negligible absorption in the visible spectral region.

**Fig. 9:**
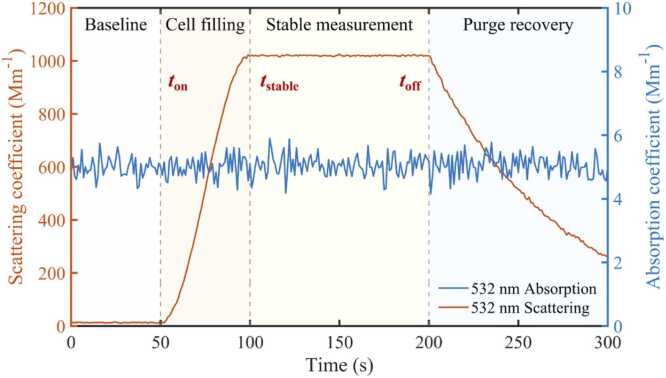
Dynamic responses of the absorption and scattering channels during water-mist introduction.

The dynamic responses are shown in Fig. 9. The vertical dashed lines mark the onset of water-mist generation ($t_{\text{on}}$), the beginning of the stable-measurement stage ($t_{\mathrm{stable}}$), and generator shutdown ($t_{\text{off}}$). The measurement process was divided into four stages: baseline, cell filling, stable measurement, and purge recovery.

During the baseline stage (t<50s), clean air was continuously introduced into the gas cell, and both the scattering and absorption channels remained at their respective baseline levels. After the water-mist generator was switched on at approximately $t_{\text{on}}\;\approx \;50\;\text{s}$, the droplets gradually entered the cell with the carrier gas, causing the particle concentration and scattering coefficient to increase continuously during the cell-filling stage from approximately 50–100 s. After approximately 50 s of filling ($t_{\mathrm{stable}}\;\approx \;100\;\text{s}$), the water-mist input, convective transport, mixing within the cell, particle wall loss, and gas outflow approached a dynamic balance. Consequently, the scattering coefficient reached a stable plateau of approximately 1000 Mm^−1^ between 100 and 200 s. After the water-mist generator was switched off at approximately $t_{\text{off}}\;\approx \;200\;\text{s}$, continuous purging gradually removed the droplets from the cell, and the scattering coefficient decreased accordingly. This dynamic behavior was primarily governed by water-mist generation, tubing transport, and gas-cell replacement.

In contrast to the pronounced response of the scattering channel, the 532 nm photoacoustic absorption channel remained near its baseline throughout the experiment and did not exhibit synchronous increasing, plateau, or decreasing behavior. As described in Section 3.2, the photoacoustic and scattering signals were acquired using independent sensing elements and signal-processing chains. Under the conditions of this strong-scattering water-mist test, the substantial change in the scattering channel did not produce a discernible synchronous response in the photoacoustic absorption channel, supporting a certain degree of channel isolation.

### 4.2 Comparison of dual-wavelength absorption responses of representative carbonaceous aerosols

Aerosols produced by cigarette smoldering and flaming combustion of absorbent cotton were separately introduced into the gas cell. The absorption coefficients at 450 and 532 nm were measured simultaneously, and the two-point AAE_450/532_ was calculated using Eq. (2) to compare the relative wavelength dependences of absorption for the two controlled-combustion aerosols.

**Fig. 10:**
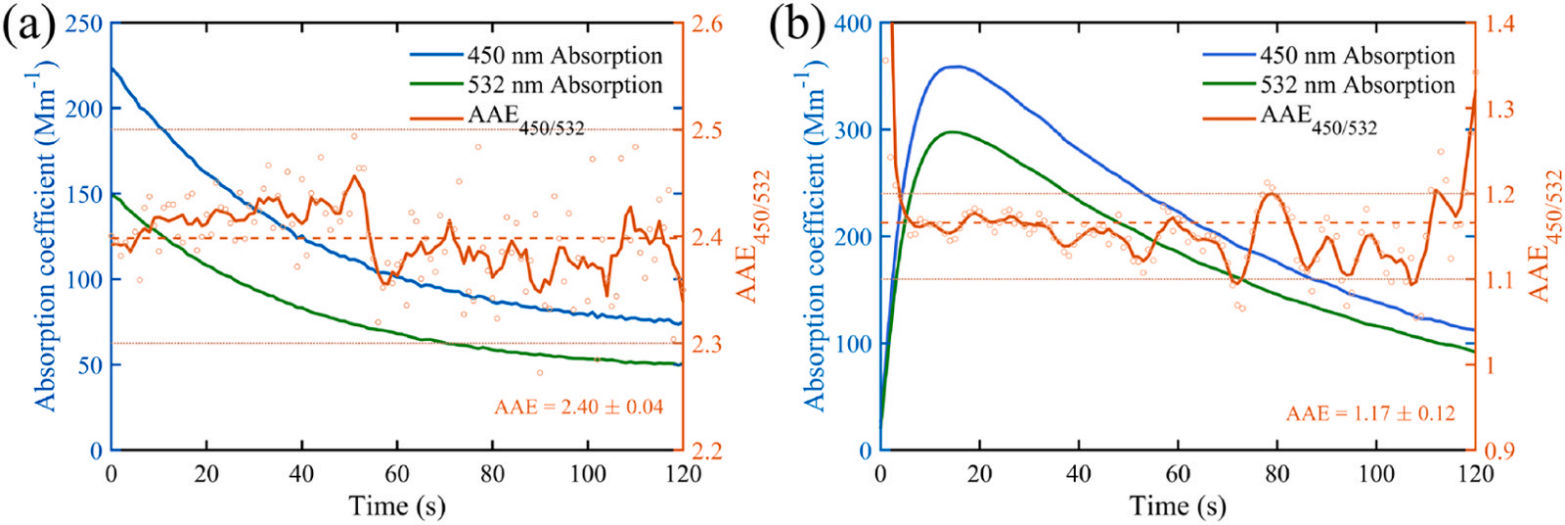
Dual-wavelength absorption coefficients and two-point AAE_450/532_ of aerosols produced by cigarette smoldering and flaming combustion of absorbent cotton: (a) cigarette-smoldering aerosol and (b) flaming absorbent-cotton aerosol.

The results for cigarette-smoldering aerosol are shown in Fig. 10(a). The 450 nm absorption coefficient remained higher than the 532 nm value, and the two channels decreased synchronously as the particle concentration decayed. The measured AAE_450/532_ was 2.40 ± 0.04, with most values distributed between 2.3 and 2.5. This result indicates pronounced short-wavelength-enhanced absorption over 450–532 nm and is generally consistent with optical characteristics associated with a relatively high contribution from organic light-absorbing components during smoldering combustion and with previous reports [42].

The results for aerosol produced by flaming combustion of absorbent cotton are shown in Fig. 10(b). The difference between the 450 and 532 nm absorption coefficients was smaller, and the mean AAE_450/532_ was 1.17 ± 0.12, indicating weaker wavelength-dependent absorption over the measured spectral range. This response more closely resembles the broadband absorption characteristics commonly associated with black-carbon-like particles. The value was slightly higher than the commonly reported value of approximately 1.0 for idealized black carbon, possibly because of contributions from organic light-absorbing components, particle morphology, or mixing state [21]. However, because chemical-composition and morphological measurements were not conducted, the underlying mechanism cannot be further determined.

The two aerosol types therefore exhibited short-wavelength-enhanced and weakly wavelength-dependent absorption, respectively, demonstrating that the system can resolve relative dual-wavelength absorption differences between controlled-combustion aerosols. Because a two-point AAE is also affected by particle composition, morphology, and mixing state, these results are not used to uniquely identify aerosol sources.

### 4.3 Atmospheric field monitoring and analysis of representative periods

**Fig. 11:**
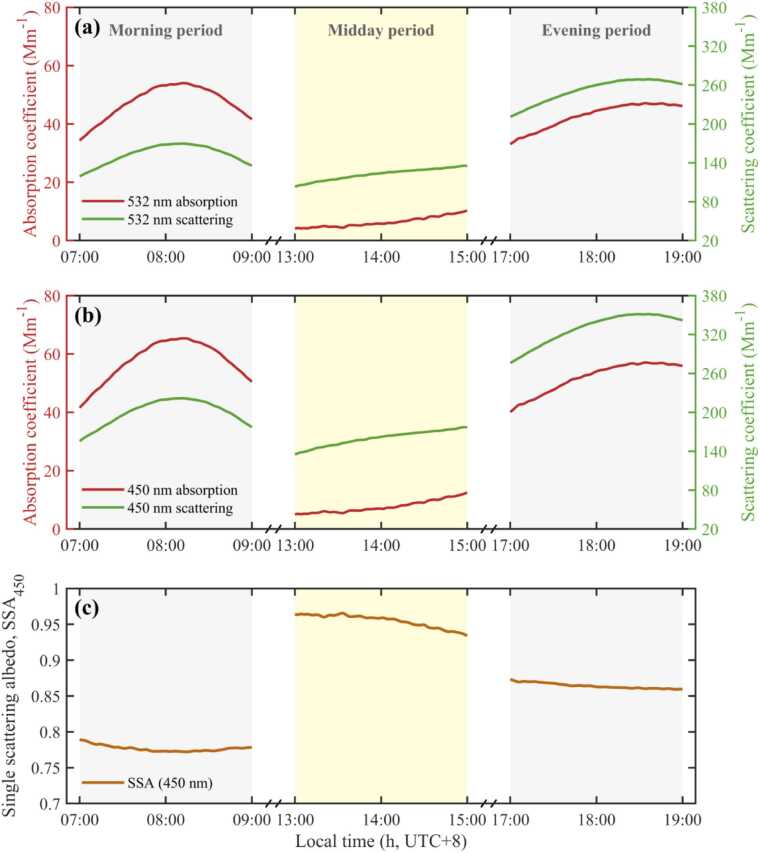
Variations in aerosol optical parameters during three representative periods of the field observation on Hefei Science Island: (a) 532 nm absorption and scattering coefficients; (b) 450 nm absorption and scattering coefficients; and (c) 450 nm single-scattering albedo.

To evaluate continuous operation under ambient conditions, the system was deployed on Hefei Science Island for online monitoring of near-surface aerosols. Fig. 11 presents the dual-wavelength absorption and scattering coefficients and the 450 nm SSA during three representative periods: 07:00–09:00, 13:00–15:00, and 17:00–19:00.

As shown in Fig. 11(a) and (b), the absorption and scattering coefficients at both wavelengths were higher during the morning and evening periods and lower around midday. The maximum 450 nm absorption and scattering coefficients were approximately 66 and 356 Mm^−1^, respectively. The morning and evening high-value periods showed some temporal correspondence with urban commuting periods and may have been jointly influenced by local anthropogenic emissions and atmospheric dispersion conditions. However, traffic volume, boundary-layer height, and particle chemical composition were not measured simultaneously; therefore, no quantitative attribution to specific emission sources is made here.

The 450 nm SSA was calculated from the synchronously measured absorption and scattering coefficients. As shown in Fig. 11(c), the 450 nm SSA was approximately 0.77–0.79, 0.92–0.96, and 0.85–0.87 during the morning, midday, and evening periods, respectively, indicating a relatively larger scattering contribution around midday. This change may be related to dilution of primary pollutants associated with daytime boundary-layer development and the formation of secondary scattering components. Nevertheless, the specific mechanisms cannot be verified without simultaneous meteorological and chemical-composition measurements. The observed 450 nm SSA range encompasses the mean value of 0.87 previously reported for Hefei [43]. Because the observation periods, locations, and measurement methods differ, this comparison is provided only as a qualitative reasonableness check.

During the observation period, the two-point AAE_450/532_ and SAE_450/532_ values were mainly distributed within 1.1–1.2 and 1.5–2.0, respectively, indicating weak wavelength-dependent absorption and pronounced short-wavelength-enhanced scattering. Because two-point Ångström exponents are influenced by particle composition, size, morphology, and mixing state, these parameters are not used to uniquely determine aerosol sources.

### 4.4 Limitations and maintenance considerations for long-term operation

The stability test conducted in this study primarily evaluated short-term drift in the acoustic detection, photon-counting, and electronic demodulation chains under controlled conditions. It does not fully represent the long-term stability of the integrating spheres after continuous aerosol exposure over several weeks or months.

As the cumulative particle loading increases, contamination of the PTFE inner surfaces may reduce the effective reflectance and scattering sensitivity while increasing the photothermal background. During practical operation, the photoacoustic zero level and PMT background count rate under clean-gas conditions should therefore be recorded periodically, and the Rayleigh-scattering calibration should be repeated using N_2_ and CO_2_. If persistent changes are observed in the scattering calibration factors or photoacoustic background, the integrating-sphere surfaces should be inspected and cleaned, followed by recalibration.

Future work will use longer exposure periods, different particle loadings, and aerosols of different compositions to quantitatively evaluate changes in PTFE reflectance, scattering-response coefficients, and photoacoustic backgrounds as functions of cumulative sampling exposure.

### 4.5 Comparison with representative photoacoustic aerosol measurement systems

To clarify the technical position of the present system, Table 2 compares representative multi-wavelength photoacoustic absorption systems and combined absorption–scattering measurement systems. Because the reported studies differ in wavelength, laser power, integration time, calibration method, and detection-limit criterion, the detection performance is not ranked solely by numerical values. The comparison instead focuses on the measured parameters, synchronization strategy, common sampling volume, and acoustic-noise suppression approach.

**Table 2:** Comparison of the present system with representative photoacoustic aerosol measurement systems.

**System and reference**	**Wavelengths**	**Measured parameters**	**Synchronization and detection approach**	**Reported detection performance**
TW-PAS, Cao et al. [22]	404, 637, and 805 nm	Three-wavelength absorption coefficients and AAE	Three acoustic resonators operating simultaneously at different resonance frequencies; single-microphone detection; no scattering measurement	Second-scale simultaneous measurements; absorption LODs of approximately 0.20–0.78 Mm⁻¹ at 200 s averaging
PAAS−4λ, Schnaiter et al. [39]	405, 473, 515, and 660 nm	Four-wavelength absorption coefficients and AAE	Four lasers coupled into a single acoustic resonator; sequential wavelength measurements; single-ended microphone detection	Basic averaging time of 5 s per wavelength; 1*σ* LODs of approximately 0.35–0.46 Mm⁻¹ at 60 s under operating conditions
PASIS-Cell, Li et al. [29]	532 nm	Absorption coefficient, scattering coefficient, and SSA	Dual T-type photoacoustic cells coupled to an integrating sphere; simultaneous single-wavelength measurements within the same sample cell	Absorption and scattering LODs both below 1 Mm⁻¹
4W-AbSc, Wang et al. [19]	405, 444, 653, and 885 nm	Multi-wavelength absorption and scattering properties	A photoacoustic resonator coupled to integrating-scattering units on both sides; combined measurements within a common sample volume	At 405 nm, absorption and scattering mass-concentration sensitivities of 3.34 and 26 *μ*g·m⁻³ , respectively
Present system	450 and 532 nm	Dual-wavelength absorption and scattering coefficients, two-point AAE and SAE, and SSA	Two spectrally separated differential acoustic modes with FDM demodulation; two symmetric microphone pairs for differential detection; a single sampling path and common measurement volume	For the 532 nm channels, 3*σ* absorption and scattering LODs of 1.2 and 1.32 Mm⁻¹ , respectively, at 1 s integration

Table 2 shows that the main characteristic of the present system is neither the largest number of detection wavelengths nor the lowest single-channel detection limit. Instead, two spectrally separated differential acoustic modes and frequency-division multiplexing are used to obtain dual-wavelength absorption and scattering parameters synchronously within a common measurement volume. Two symmetric microphone pairs provide differential detection, and the optical parameters required to calculate AAE, SAE, and SSA are acquired through a single sampling path. The principal features of the architecture are therefore multiparameter synchronization, a common measurement volume, and suppression of in-phase common-mode disturbances.

The present study did not include simultaneous comparisons with commercial absorption and scattering instruments or a conventional split-type system. Therefore, no quantitative conclusions are drawn regarding the absolute measurement bias for real aerosols or the extent to which the common-volume architecture reduces spatiotemporal non-homogeneity bias. Future work will include parallel measurements with reference instruments and controlled humidity and particle-transport experiments to evaluate the measurement accuracy and practical advantages of the integrated architecture.

## 5 Conclusion

A spatiotemporally synchronized dual-wavelength aerosol absorption–scattering measurement system based on a dual-differential sphere–tube coupled gas cell was developed. The two integrating spheres simultaneously serve as scattered-light collection cavities, acoustic buffer chambers, and components of a low-frequency differential Helmholtz resonator, while the central acoustic tube supports a high-frequency differential second-order longitudinal standing-wave mode. Combined with frequency-division multiplexing and FPGA-based digital lock-in demodulation, this configuration enables simultaneous measurements of the 450 and 532 nm absorption and scattering coefficients through a single sampling path and within a common measurement volume.

The two photoacoustic absorption channels were calibrated using NO_2_ standard gas, and the two scattering channels were calibrated using a two-point N_2_/CO_2_ Rayleigh-scattering method. At an integration time of 1 s and using a 3*σ* criterion, the limits of detection for the 532 nm absorption and scattering channels were 1.2 and 1.32 Mm^−1^, respectively. During the water-mist experiment, the pronounced change in the scattering channel did not produce a discernible synchronous response in the 532 nm absorption channel. Aerosols produced by cigarette smoldering and flaming combustion of absorbent cotton exhibited different two-point AAE characteristics, demonstrating the capability of the system to measure relative dual-wavelength absorption differences. The field observations further demonstrated the feasibility of continuously acquiring dual-wavelength optical parameters and the 450 nm SSA under ambient conditions.

The present study did not include simultaneous comparisons with commercial absorption and scattering instruments, a conventional split-type system, or a single-ended photoacoustic detection configuration. Therefore, the current results are not used to quantify the absolute measurement bias for real aerosols, the reduction in spatiotemporal non-homogeneity bias provided by the common-volume architecture, or the signal-to-noise-ratio improvement achieved by differential detection. Future work will include parallel measurements with reference instruments, controlled humidity and particle-transport experiments, and a comprehensive evaluation of measurement accuracy and uncertainty.

## CRediT authorship contribution statement

**Zhengang Li:** Writing – original draft, Validation, Methodology, Formal analysis, Data curation. **Hepeng Zhang:** Writing – original draft. **Daming Dong:** Investigation, Conceptualization. **Yonghua Fang:** Supervision, Project administration.

## Declaration of Competing Interest

The authors declare that they have no known competing financial interests or personal relationships that could have appeared to influence the work reported in this paper.

## Data Availability

Data will be made available on request.
